# Supplementary material for: Yiai Fuzheng decoction inhibits triple-negative breast cancer by remodeling the immune microenvironment
Source: Front Immunol. 2025 Sep 30;16:1615631. doi: 10.3389/fimmu.2025.1615631 (PMC12518410; doi:10.3389/fimmu.2025.1615631)
Supplement: Supplementary file 2 [file DataSheet1.zip › Supplementary File 2/Treatment/C_vs_D/07_Pathway_Analysis/Pathway_Analysis/Sig_Pathways_Name/Pyrimidine metabolism.pdf]

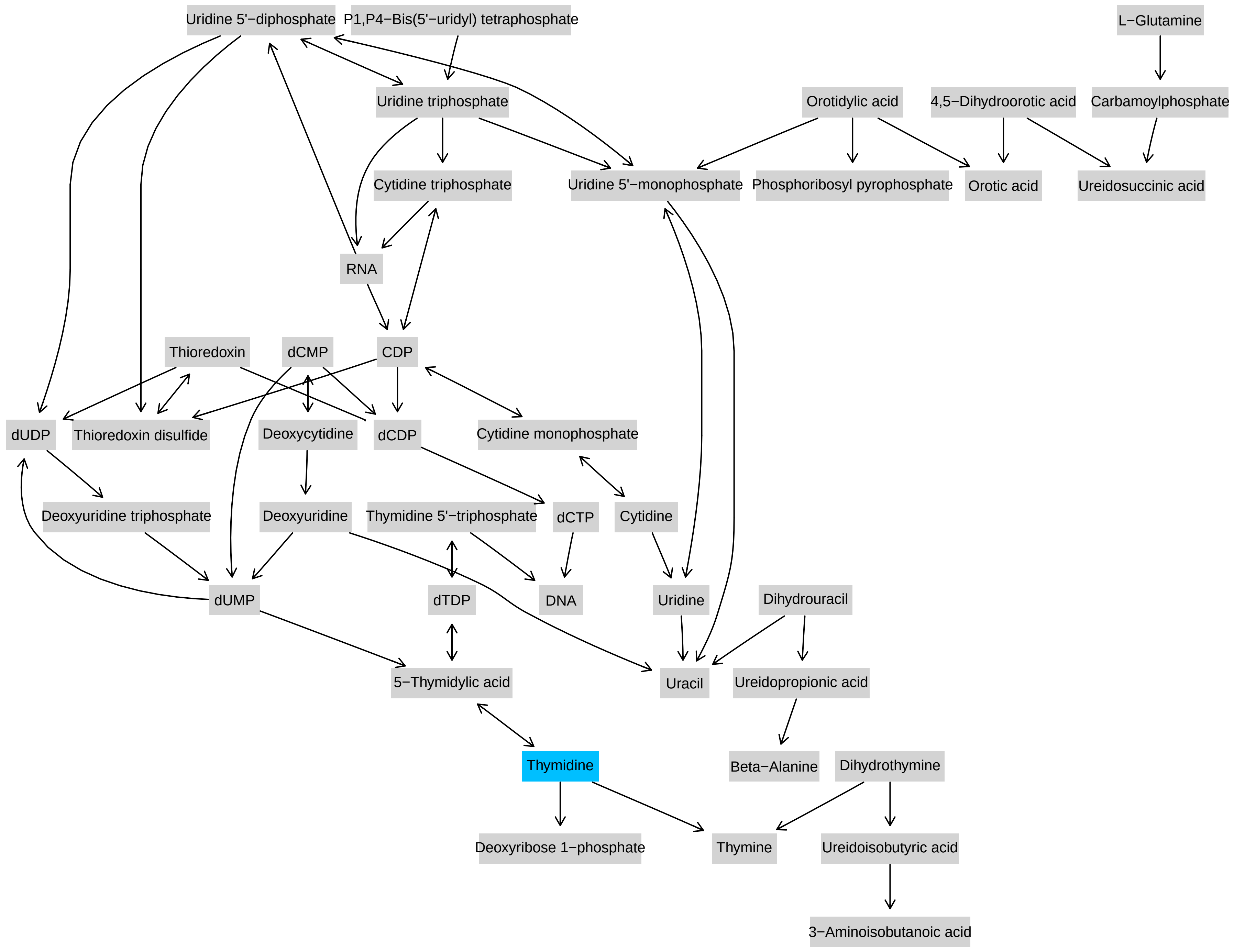
Uridine 5'−diphosphate
P1,P4−Bis(5'−uridyl) tetraphosphate
L−Glutamine
Uridine triphosphate
Orotidylic acid
4,5−Dihydroorotic acid
Carbamoylphosphate
Cytidine triphosphate
Uridine 5'−monophosphate
Phosphoribosyl pyrophosphate
Orotic acid
Ureidosuccinic acid
RNA
Thioredoxin
dCMP
CDP
dUDP
Thioredoxin disulfide
Deoxycytidine
dCDP
Cytidine monophosphate
Deoxyuridine triphosphate
Deoxyuridine
Thymidine 5'−triphosphate
dCTP
Cytidine
dUMP
dTDP
DNA
Uridine
Dihydrouracil
5−Thymidylic acid
Uracil
Ureidopropionic acid
Thymidine
Beta−Alanine
Dihydrothymine
Deoxyribose 1−phosphate
Thymine
Ureidoisobutyric acid
3−Aminoisobutanoic acid
